# Implementation of a Novel Multi‐Center Pediatric Liver Transplant Collaborative Network

**DOI:** 10.1111/petr.70530

**Published:** 2026-11-03

**Authors:** Kyle A. Soltys, Regino Gonzalez‐Peralta, Robert Teaster, Nicolas Goldarecena, Frank DiPaola, Shawn Pelletier, Kenneth Brayman, Michael Angelis, L. Thomas Chin, Ajai Khanna, Noel Thompson, Rakesh Sindhi, Barry Friedman, David Levi, Daniela Damian, Raj Aneja, Diane Hupp, Sara Rasmussen, George Mazariegos

**Affiliations:** ^1^ Hillman Center for Pediatric Transplantation Thomas E. Starzl Transplantation Institute, UPMC Children's Hospital of Pittsburgh Pittsburgh Pennsylvania USA; ^2^ Advent Health Transplant Institution, Adventhealth for Children Orlando Florida USA; ^3^ University of Virginia Medical Center Charlottesville Virginia USA; ^4^ Department of Surgery Hospital for Sick Children Toronto Ontario Canada; ^5^ Guidry and East LLC Fayetteville Arkansas USA; ^6^ AtriumHealth, Wake Forest School of Medicine Charlotte North Carolina USA; ^7^ UPMC Children's Hospital of Pittsburgh Pittsburgh Pennsylvania USA; ^8^ Nationwide Children's Hospital Columbus Ohio USA

**Keywords:** failure to rescue, learning network, living donor liver transplant, multi‐institutional network, pediatric liver transplantation, quality assurance and performance improvement, split liver transplant, technical variant transplantation

## Abstract

**Background:**

Initiation or augmentation of pediatric liver transplant programs in the United States faces challenges of limited expertise, small volumes, and regional variation in access to care. PACH launched a prospective plan of pediatric liver program development at two sites (VAUV and FLFH) with the goals of comprehensive multidisciplinary program support, consistent surgical leadership from one team, consensus protocol development, and standardization of perioperative and postoperative care, and comprehensive long‐term follow‐up care plans. A third center, NCCM, was launched in June 2024.

**Methods:**

Between January 2016 and December 31, 2024, 140 children received pediatric liver transplants at one of the collaborative sites. A total of 215 PLT were performed at PACH during the same period.

**Results:**

Patient and graft survival were 94.4% and 93.2% respectively, with current follow‐up of 1674 ± 1037 days. Overall patient demographics, complications, and patient outcomes were similar among all institutions.

**Conclusions:**

A paradigm of systematically extending center experience directly to facilitate safe and high‐quality pediatric liver program establishment has been demonstrated, with the additional benefit of increased split liver graft utilization benefiting both adults and children. The success of this model has implications for optimizing pediatric liver transplant care in the United States.

## Graphical abstract

Collaborative program development. Each program was developed in successive steps from initial education and protocol development with established punch lists and metrics for program launch. Once the program was launched with onsite and virtual support, regular operations meetings of all clinical and administrative team members afforded necessary protocol management and establishment of quality initiatives. All phases of transplant are done as a collaborative team—from patient evaluation, listing, care while waiting for transplant, and the perioperative phase. As the local experience increases and meets mutually decided‐upon metrics, onsite support of all but surgical staff diminishes. The final phase of the collaborative program development involves data sharing and quality projects with participation in the Starzl Network for Excellence in Pediatric Transplantation with development of robust cross‐institutional quality projects.

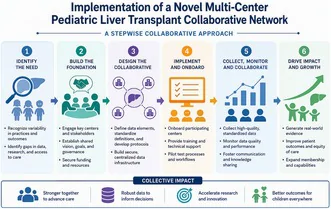

**Abbreviations**
CMS: centers for medicare and medicaid services
FLFH: adventhealth, Orlando, Florida
HAT: hepatic arterial thrombosis
HVS: hepatic vein stenoses
NCCM: atrium health, Charlotte, North Carolina
PACH UPMC: Children's Hospital of Pittsburgh
PELD: pediatric end‐stage liver disease
PLT: pediatric liver transplant
PTLD: post‐transplant lymphoproliferative disease
PVS: portal vein stenoses
PVT: portal vein thrombosis
QAPI: quality assurance and performance improvement
SBO: small bowel obstruction
SNEPT: starzl network for excellence in pediatric transplantation
SPLIT: society of pediatric liver transplantation
UNOS: united network for organ sharing
VAUV: university of virginia health sciences center, Charlottesville, Virginia

## 1 Introduction

Approximately 500 pediatric liver transplants (PLT) are performed annually in the United States [1], and while 90 institutions have been approved to perform PLT under the specific 2021 CMS pediatric guidelines, only 51 performed one or more PLT that year. Despite the guidelines, variability remains in center volumes, with seven centers performing 20–39 cases, 13 centers performing less than 10 and 18 centers performing less than 5 cases [1]. Significant variability in center graft and patient survival after PLT prompted the development of qualifications for centers to be allowed to perform transplants in children. These guidelines focus on cente‐specific metrics and the minimum center surgeon and physician experience thought to impact survival in a cohort of children undergoing liver transplant in 2011 [2]. However, these program requirements were limited to the primary surgeon and hepatologist of the respective programs and had limited impact on the quality or volume of PLT performed after the introduction of the criteria.

Center variability also exists in the pretransplant survival of children on the waitlist. Despite the application of living donor liver transplant and well‐known techniques for the use of other technical variant liver allografts in children, more than 5% of small children continue to die or become too sick to undergo transplantation [3]. There remains an unacceptably high difference in the application of technical variant expertise, which has been shown to impact waitlist mortality [2].

To address these challenges, we initiated discussions with institutions to create collaborative pediatric liver transplant teams and codevelop dedicated, pediatric‐specific protocols and processes to deliver expert care to children in need of PLT. This report discusses the processes used to develop these partnerships and presents data on our initial cohort of patients at the collaborative sites.

## 2 Materials and Methods

Program development and implementation consisted of three phases as shown in Figure 1.

**FIGURE 1:**
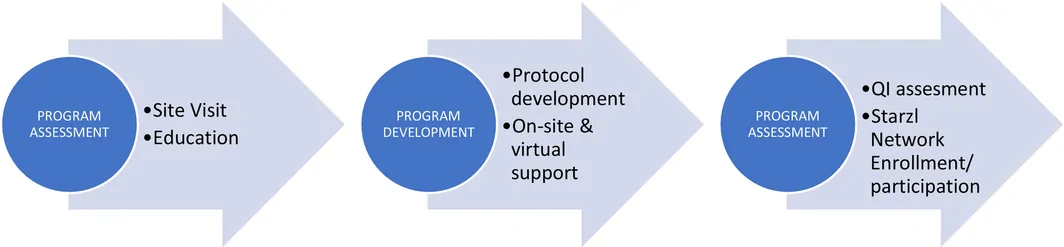
Components of program development. Each program was developed in three successive steps, from initial education and protocol development with established punch lists and metrics for program launch. Once the program was launched with onsite and virtual support, regular operations meetings of all clinical and administrative team members afforded necessary protocol management and establishment of quality initiatives. The final phase involves data sharing and quality projects, with participation in the Starzl Network for Excellence in Pediatric Transplantation and ongoing learning within the collaborative.

Stage 1 consisted of preliminary site visits and assessment of existing program infrastructure and multidisciplinary expertise. This 12‐to‐18‐month process allowed for an understanding of the services immediately available, as well as any gaps in infrastructure or services at individual centers. This stage culminated in the development of a professional services agreement between participating institutions. Stage 2 consisted of virtual and onsite nursing education, protocol codevelopment and completion of licensing and credentialing requirements for the required team members. Protocols were developed by a combined team from participating institutions to individualize preexisting protocols from UPMC Children's Hospital of Pittsburgh (PACH) for the different centers. High‐level immunosuppressive, anticoagulation and infectious disease protocols were standardized among centers. Educational programs were carried out, and individual services (radiology, laboratory, and genetics) were involved in breakout sessions, including mock transplant procedures. During the program setup phase, guidelines were initiated for referrals, recipient selection, donor evaluation, surgical and medical team mobilization, and immediate and long‐term posttransplant follow‐up. Protocols were also developed to manage common complications of PLT, and care plans were developed with interventional radiology, ultrasound, and other complication‐specific services. Program launch was done only after completion of collaborative “punch‐lists”. Once the programs became active, *onsite* critical care, anesthesia, nursing, and surgical staff from PACH partnered with the staff at the institutions at the time of each PLT. Candidate evaluations were done collaboratively with the local teams, and PACH surgeons met with all candidates and their families at the time of the local surgical team visit. Listing conferences and routine list management also occurred collaboratively. All donor offers (including living donors) were reviewed as a group, and the logistics of each case were developed in real time by members of the teams. Over the course of this phase, specific and jointly determined proficiencies were demonstrated by the centers, and the participation of supporting staff in the onsite care of patients was reduced. *Onsite* surgical support at the time of transplant remained for the duration of the collaboration. Clinical and program assessment phases consisted of weekly multidisciplinary clinical team meetings, daily inpatient virtual rounds, quarterly virtual leadership meetings, and quality dashboard reviews. PACH surgeons rounded daily using videoconferencing platforms and remote electronic medical record access on review all inpatients and address any urgent outpatient issues. During Phase 3, additional program component expertise was developed, as well as program review in QAPI forums locally and in a shared multicenter forum, allowed for collaborative assessment of quality metrics, such as the time from referral to listing, as well as other program‐specific metrics. Finally, participation in the Starzl Network for Excellence in Pediatric Transplantation (SNEPT) learning health collaborative facilitated transparency of results, sharing of practices, and ongoing learning from the network.

## 3 Statistical Analysis

Comparison of groups was performed by Fisher's exact test. Significance was assumed at a *p*‐value of < 0.05. Time‐to‐event outcomes were compared between groups using Kaplan–Meier methods and Cox proportional hazards regression. A cox proportional hazards model was utilized to compare groups, generate 95% confidence intervals, and establish hazard ratios.

## 4 Results

Between 5/1/2016 and 12/31/2025, the PLT team from UPMC Children's Hospital of Pittsburgh participated in 355 PLTs (215 PACH, 50 VAUV, 75 FLFH, 11 NCCM). Patient and center demographics are shown in Table 1. Collaborative sites were combined as a single group for comparison with patients and results at PACH. There were 11 patients transplanted for acute liver failure at the collaborative sites as status 1A, 33 patients transplanted as status 1B (7 hepatoblastoma, 7 chronic liver disease, and 22 metabolic), two retransplants with primary LT performed elsewhere, and one patient who was retransplanted for chronic rejection 996 days after her primary transplant. Mean allocation PELD score at network sites was 34.8 ± 10.8, and 72% of non‐status 1 patients were transplanted with written, nonstandard exceptions. Only 30 network patients were transplanted with their calculated scores. Indications for transplant differed significantly between the two groups of patients, with more transplants performed for metabolic disease at PACH. Biliary atresia and nonclassified cirrhosis were more common at the collaborative sites. Overall patient and graft survival were 94.4% and 93.2%, respectively, with a mean follow‐up of 1652 ± 1036 days. All patients who died had functioning grafts.

**TABLE 1:** Demographics, diagnoses and liver graft types used in 355 children undergoing liver transplant.

	*N*	%	
Diagnosis (regional sites)			
EHBA	60	43	
Metabolic	26	19	
ALF	11	8	
Other cirrhotic	32	23	
Tumor	7	5	
Re‐transplant	4	3	
Graft type (regional sites)			
Whole	40	29	
TVG	100	71.4	
LDLT	16	11	
Demographics (regional sites)			
Mean age (months)	73 ± 72		
Mean weight (kg)	23.7 ± 20.6		

	*N*	%	*p*
Diagnosis (PACH)			
EHBA	48	22	< 0.001
Metabolic	108	50	< 0.001
ALF	9	4	0.162
Other cirrhotic	20	9	< 0.001
Tumor	21	9.8	0.11
Re‐transplant	9	4.2	0.38
Graft type (CHP)			
Whole	44	20	0.096
TVG	171	79.5	0.096
LDLT	83	39	< 0.001
Demographics (CHP)			
Mean age (months)	53 ± 51	0.06	
Mean weight (kg)	20.2 ± 17	0.08	

### 4.1 Graft Type Utilization

Technical variant allografts represented 74% of all livers utilized across the entire cohort of patients. Living donors represented more than a third of allografts at CHP, significantly more than at the regional network sites. We began using living donor livers at the University of Virginia in 2022, and of the 25 LT since that time, 16 (64%) of cases have used left lateral segment living donor allografts. Interestingly, 83% of the LD were from anonymous, altruistic donors. When deceased technical allografts were utilized, the primary adult donors allocated to our pediatric population as a split resulted in the transplantation of an adult recipient with an extended right lobe graft in 58 cases. Secondary patient and graft survival of the extended right lobe graft in cases performed at our collaborative centers was 95.5% and 95.5%, respectively. In addition, three patients with maple syrup urine disease were successful domino liver donors to three adults on the corresponding center's waitlist. During the same period, 15 domino liver transplants were performed at PACH. Overall, for the 140 pediatric liver transplants performed at the collaborative sites, only 40 were performed as a whole liver allograft. Allograft reduction instead of split liver recovery was performed in 34 patients who required transplant of the vena cava due to tumor proximity or due to the recipient requiring the volume of a left lobe allograft from an adult or pediatric donor. All other reductions were performed because the extended right allograft could not be placed.

### 4.2 Post Transplant Complications

Post‐operative complications were similar across all sites. For purposes of the manuscript, post‐operative hepatic arterial thrombosis (HAT) was considered to be those occurring beyond the exit from the operating room and was recorded separately from intraoperative arterial thromboses (iHAT). Intraoperative thromboses were treated with immediate operative thrombectomy and thrombolysis and revision of the arterial anastomosis when indicated. Early post‐operative HAT underwent emergent exploration and attempted re‐arterialization. Portal vein stenoses (PVS) were managed with balloon venoplasty without stenting, while portal thrombosis (PVT) was managed conservatively unless there was evidence of varices or hepatopulmonary syndrome, which were managed with creation of a mesorex bypass in four patients. One mesorex bypass at PACH was unfortunately damaged during an exploration for an adhesive small bowel obstruction 16 days after it was created. Other post‐transplant complications included three hepatic vein stenoses (HVS) that required intervention balloon dilatation (one was re‐transplant patient from another center and required deployment of an expandable stent). There was a 17% incidence of biliary complications (12% at the collaborative sites vs. 17.2% at PACH) requiring percutaneous or endoscopic manipulation across the strictures. Three patients with recurrent biliary stricture required operative reconstruction. Five patients had explorations for bowel obstructions (SBO), and three patients had early post‐operative intestinal perforations, one from the jejunal‐jejunal anastomosis. Post‐transplant lymphoproliferative disease (PTLD) was rare, did not cause any mortality or graft loss, and occurred at a similar rate between the groups. Complication rates were not found to be significantly different between the network sites and PACH (Table 2).

**TABLE 2:** Comparison of complications in 355 children undergoing liver transplant across the network.

Complication (regional sites)			
	*N*	%	
Biliary	17	12	
iHAT	1	0.7	
HAT	3	0.7	
PVS	5	3.6	
PVT	1	0.7	
HVS	2	1.4	
SBO	2	1.4	
Perforation	1	0.7	
PTLD	2	0.14	

	*N*	%	*p*
Complication (CHP)			
Biliary	37	17	0.23
iHAT	3	1.4	0.98
HAT	5	2.3	0.41
PVS	9	4.2	0.98
PVT	5	2.3	0.41
HVS	1	0.5	0.56
SBO	3	1.4	0.98
Perforation	2	0.9	0.65
PTLD	7	3.3	0.49

### 4.3 Graft and Patient Survival

Kaplan–Meier survival curves (Figures 2 and 3) demonstrated similar survival trajectories between the groups across the duration of follow‐up. Formal comparison using a two‐sided log‐rank test did not demonstrate a statistically significant difference in either graft or patient survival between groups. To further quantify the association between group assignment and risk of an event, a Cox proportional hazards model was fit with group as the sole covariate, with the regional network group as a reference, and was associated with a hazard ratio of approximately 1.1, with a 95% confidence interval that included unity and a non‐significant Wald test (*p* > 0.05).

**FIGURE 2:**
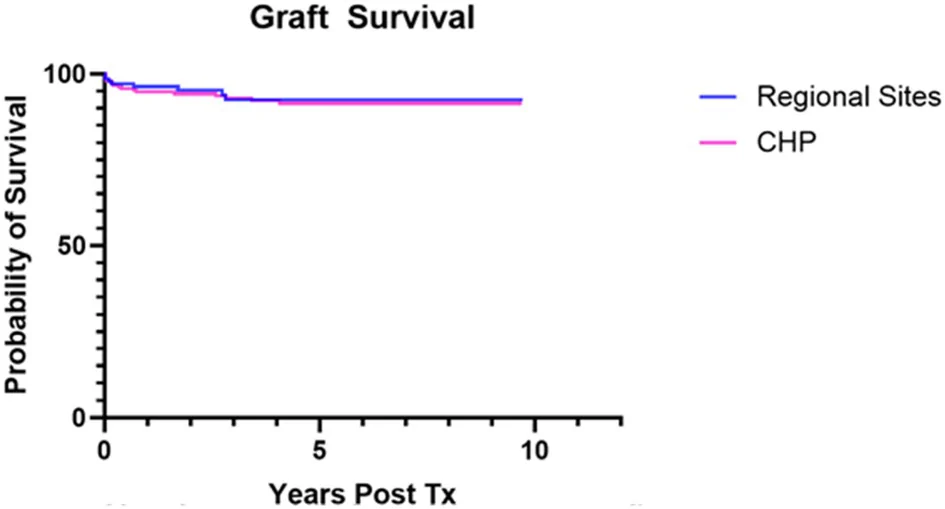
Kaplan–Meier curve comparing graft survival at collaborative network sites in comparison with patients transplanted at PACH.

**FIGURE 3:**
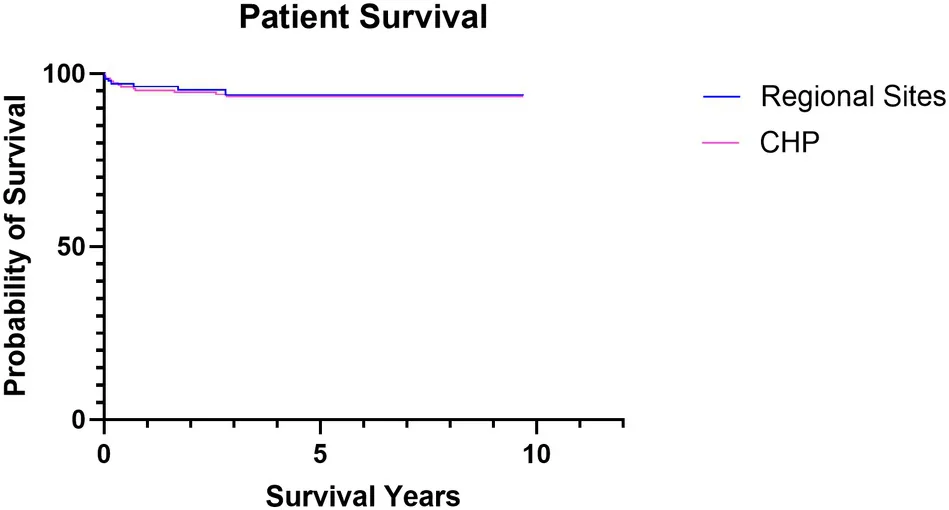
Kaplan–Meier curve comparing patient survival at collaborative network sites in comparison with patients transplanted at PACH.

## 5 Discussion

Disparity in pediatric LT access and outcomes has been well documented in the United States [4]. Although recognition of this issue led UNOS to create requirements for centers to perform pediatric liver transplants, it did not offer any potential solution to help centers determine the reasons behind the disparity or improve upon their results. As the number of patients is relatively small, consistent expertise to care for children suffering from unresectable tumors, end‐stage liver disease, and rare metabolic diseases is lacking around the country. As a result, children often do not have local access to expert care and may face a lack of full transparency in relation to the full range of options they have and their expected outcomes. Although surgical expertise is a critical variable influencing survival, appreciation for the broad infrastructure required, including hepatology, critical care, nursing, and the system requirements to optimize outcomes, has not been formally studied outside of demonstration of significant differences in both outcomes [5] and failure to rescue [6].

Institutions that desire to optimize care for pediatric LT patients have limited options to serve a very small group of patients: they can invest in developing a specialized pediatric LT program from the ground up or attempt to assign local expertise in PLT (usually adult liver transplant teams) to a low volume, high risk patient population. Both approaches face a significant learning curve and require significant resources. In addition, small volume, low‐performing centers are less likely to be able to care for complex and more high‐risk children, utilize technical variant allografts, and manage the complications associated with those transplants with resultant increases in pre‐ and post‐transplant morbidity, rescue failure, and mortality. Of equal importance, patients and families often face significant geographic, financial, and insurance barriers and other disparities that make traveling to national high‐volume and higher‐performing centers very challenging [4].

This gap in care led us to develop a multi‐state collaborative network that overcomes the disparity in center experience by providing an experienced team of surgeons, physicians, nurses, and other support staff to modify practices at institutions that were not performing large (or any) volumes of PLT. The level of experience within the group at UPMC Children's Hospital of Pittsburgh is relatively unique in the United States, with all staff surgeons having participated in more than 500 pediatric liver transplants during their careers. As a direct result of the center experience, pediatric hepatology, nursing, anesthesia, critical care, and other subspecialties have a similar depth of experience in managing children awaiting and following PLT. As described above, implementation of these programs required periods of education and protocol development, but once the programs were started, highly complex children were immediately transplanted with excellent waitlist and post‐transplant survival and perioperative morbidity. Introduction of larger‐volume pediatric liver transplant programs at sites that had limited experience in the field prior to the creation of these programs led to exposure of a rare specialty to residents and fellows who had little to no prior exposure. Indeed, one of the ASTS multiorgan fellows from one of these sites spent an extra year at UPMC Children's Hospital of Pittsburgh for in‐depth pediatric transplant training.

Post‐transplant complications were limited, despite the large number of technical variant allografts used to transplant very ill patients. Patient management was streamlined with rapid recognition and multidisciplinary involvement in complications, which was particularly important in patients with biliary strictures that require percutaneous or invasive endoscopic management. Discussion of these complications and development of an algorithm for their management were a vital part of the initial phase of program development and were part of mock patient scenarios at each institution. This preparation led to a very high rescue rate, which has been shown to be a useful metric in multiple surgical specialties, including pediatric liver transplant [6].

In addition to the impact these programs had on the institutions, augmentation of split liver transplantation in this network significantly benefited both adults and children as described by Vargas et al. [7]. The utilization of significant numbers of technical variant livers allocated to children for two recipients is in line with the demonstrated efficacy of these grafts for both the pediatric and the adult recipient [8]. The team proficiency in split graft recovery, as well as the personnel availability to perform simultaneous adult and pediatric technical variant transplants, was important to the success of the transplants. This process was the result of intensive multidisciplinary planning and was also utilized for successful domino and living donor liver transplants. Machine perfusion was utilized to facilitate the utilization of both the adult extended right segmental graft for logistic reasons or for graft optimization and included ex vivo split after machine perfusion [9, 10].

Development of robust quality improvement tracking methods, as well as incorporation into national pediatric liver transplant communities such as the Society of Pediatric Liver Transplantation (SPLIT) and the Starzl Network, facilitated ongoing education, quality improvement, and opportunities for participation in patient‐reported outcomes [11, 12].

## 6 Conclusion

Since the first liver transplant 60 years ago, pediatric liver transplant has developed into a true *subspecialty* with an explosion of information regarding donor utilization, technical aspects of transplantation and management of complications, immunosuppression, and long‐term management. All phases of transplantation—from evaluation to the perioperative and long‐term time points—require very specialized knowledge to optimize short and long‐term outcomes. Relegation of the care for these complex patients to inexperienced teams or as a part‐time focus likely accounts for much of the variability in outcomes among centers in the United States. This report describes the development and results of our unique multicenter collaborative network, something that has not been described in the past.

In developing collaborative center protocols, we understood that success would hinge on proper timing and execution of evaluations, waitlist and pre‐transplant medical management, utilization of optimal allograft types, avoidance of technical complications, perioperative management, and careful long‐term follow‐up. Direct involvement of the team in all phases was critical, as pre‐ and peri‐operative management of these patients can dramatically change post‐operative outcomes. Long‐term follow‐up of these children is also critical, with rapid implementation of weaning protocols, as well as the development of comprehensive transition programs as the cohort ages.

The collaborative network approach offers a potential method to initiate or potentially consolidate pediatric liver transplant programs in the United States, reducing the complexity of identification and onboarding of new team members and access to rare expertise. This approach eliminates any learning curve by bringing an established team, with its pre‐existing level of experience, to the site to essentially expand the on‐site team. It allowed utilization of all types of allografts, including split, living donor, and domino allografts with excellent results, despite the very sick pediatric candidates in this cohort.

Although our approach is labor intensive, the paradigm provides an expert level of care while building pediatric transplant expertise in the respective hospital teams and eliminates the burden families experience in traveling long distances to receive care at a high‐performing center. Our approach has fostered collaboration rather than competition. While the support from all participating sites is significant, the results have prompted great interest in further expanding this network and offering a new paradigm for the performance of pediatric liver transplantation.

## Data Availability

The data that support the findings of this study are available from the corresponding author upon reasonable request.
